# Book Reviews

**Published:** 1829-08

**Authors:** 


### CRITICAL ANALYSES.

Quæ laudanda forent, et quæ culpanda, vicissim

Illa, prius, cretâ; mox hæc, carbone, *notamus.*—PERSIUS.

*A Pathological Inquiry into the secondary Effects of Inflammation of the Veins.* By JAMES M. ARNOTT, Surgeon. (From the fifteenth Volume of the Medico-Chirurgical Transactions, published by the Medical and Chirurgical Society of London.—8vo. pp. 131. 1829.

WE have, upon more than one occasion, declined entering upon the subject of venous inflammation, until the present very valuable pathological contribution of Mr. ARNOTT's came under our notice. We shall now give a copious abstract of his paper.

It is not necessary to preface our analysis by formally pointing out the importance of the subject. The numerous and curious facts which have lately been given to the profession relating to the origin and progress of phlebitis, and the obscurity that hangs over the pathology of the disease, are well known to every medical reader, and must have excited his attention. It is true that the subject has been frequently touched upon by various able writers and acute observers, but still we were much in need of a succinct and satisfactory collection of cases, and of a complete revision of former opinions respecting the pathology of venous inflammation and its consequences; for the present investigation will show that the doctrines maintained, even but a few years ago, upon this very important point, were, if not altogether, at least to a great extent, founded upon erroneous views and a superficial examination of the subject.

Considerable doubt still seems to prevail as to the cause of the alarming constitutional affection frequently attendant on phlebitis, and much obscurity exists as to the origin of those abscesses and inflammations in distant parts, which sometimes occur after injuries. Mr. Arnott conceives that "an attempt to remove the one, and to dispel a portion of the other, may not therefore be considered as altogether unworthy of notice."

The attention of Mr. A. was particularly directed to this subject by some occurrences which marked the course and termination of three fatal cases of inflammation of the veins after venesection, which he had an opportunity of observing.

"In one of these, a deposition of pus, without signs of previous inflammation, took place under the skin of the opposite forearm; in another, destructive inflammation of the knee-joint, with a deposition of pus into the cellular substance of the thigh; and in the third, collections of matter at several points in the substance of the lungs; while, in all three, the inflammation of the vein did not extend to the heart. These circumstances suggested inferences as to the cause and nature of the constitutional affection in cases of phlebitis, and views with regard to the origin of abscesses in remote situations arising from injuries, which led to an examination of the evidence on both these subjects, to be found in the writings and observations of others." (P. 1.)

As we intend to confine ourselves principally to the opinions entertained by Mr. Arnott, we shall pass rapidly over the retrospective view he takes of the statements of previous writers upon the subject, which it was, however, very necessary he should introduce for the purpose of showing he had imagined no discrepancy of opinion that did not really exist. Mr. HUNTER, in his original essay on Inflammation of the Internal Coats of Veins, threw out two suggestions as to the manner in which this affection might influence the constitution. He remarks that, "in all cases where inflammation of veins runs high, or extends itself considerably, *it is to be expected* that the whole system will be affected. For the most part, the same kind of affection takes place which arises from other inflammations, with this exception, that, where no adhesions of the sides of the vein are formed, or where such adhesions are incomplete, pus, passing into the circulation, may add to the general disorder, and even render it fatal."* Mr. Hunter likewise supposed that death might arise from the inflammation of the vein extending to the heart.

Mr. HODGSON† states that the inflammation extends in

* Influenced by this view of the circumstances which favor the occurrence of alarming symptoms in cases of phlebitis, Mr. Hunter in one case applied pressure above the wounded portion of the vein, so as to bring the sides of the vessel into contact, and to cause their adhesion. This case terminated successfully.—*Transactions of a Society for the Improvement of Med. and Chir. Knowledge*, vol. i. p. 29.—REV.

† On Diseases of Arteries and Veins, London, 1815, p. 511.

some instances even to the membrane which lines the cavity of the heart, and he describes the symptoms which attend the disease as having a striking resemblance to those of typhous fever. Mr. H. considers that the constitutional irritation may be an effect produced upon the nervous system by the pus which is secreted into the vessel being mixed with the circulating blood. Although Mr. Hodgson was aware of the very formidable character of the symptoms dependent upon phlebitis, we believe he does not point out the various organic lesions which so frequently follow, and respecting which Mr. Arnott has given us so much useful information.

Mr. Travers* distinguishes the cases where inflammation of the vein terminates in the formation of pus, and where it terminates in the deposition of adhesive matter or lymph; the latter extending to the trunks of the system, and sometimes, it is said, reaching the heart. He observes, there is a marked difference in the symptoms accompanying these states: the first is a protracted irritation, producing hectic, and ending in exhaustion; the second is a typhoid fever, which, speedily producing delirium, terminates within a few days. The former cases, although always dangerous, are sometimes recovered; the latter, he imagines, never. Mr. T. then proceeds,

"There have been many conjectures respecting the cause of the fatal termination of these cases, at which I confess I feel surprised: among others, the inflammations by extension to the heart, or the membranes of the brain, and the conveyance of pus into the circulation, have been mentioned. Not to insist on the innocuous quality of pus, it should be observed, that the most rapidly destructive inflammation is that which has the true adhesive progress, in which no pus is secreted. But, if we consider the importance of the veins in the economy, the extent of surface which the collective areæ of the venous trunks afford, (larger, I imagine, than any of the shut sacs of the body,) and the diffused and disorganizing character of the inflammation, we can surely be at no loss to account for the disturbance of the system. It is an error to suppose that any quicker sympathy exists between the constitution and the venous, than the arterial or absorbent, system. I say this, because I have observed something like that superstitious alarm which is excited by events that we do not expect, and cannot explain, has been produced by the fatal catalogue of tied veins, and a comparison of this with the generally successful cases of tied arteries. All the mystery of veins is, as I have attempted to show, that they are indisposed to inflame, but, when excited,

* Essay on Wounds and Ligatures of Veins, in Cooper and Travers' Surgical Essays, vol. i. third edition, p. 236. London, 1818.

inflame by continuity, and therefore it is that the constitution sympathises so deeply."* (P. 5.)

Mr. Guthrie, who observed the veins inflamed after amputation, in connexion with, or, as he believes, as a consequence of a diseased condition of the stump, assumes† that inflammation of the veins is of two kinds, viz. the adhesive or healthy, and the irritative, erysipelatous, or unhealthy. The first kind is represented as seldom observed, but, when observed, is usually cured; the latter is almost as invariably fatal, but in what manner he offers no explanation.

Sir Astley Cooper‡ asserts that sometimes life is destroyed by inflammation extending to the large vein and to the heart. He also remarks, that if the matter resulting from the suppurative inflammation does not point, but remains in the veins, it produces excessive constitutional irritation, which destroys life.

The opinions of Breschet, Bouillard, and Ribes, are also referred to.

It appears, then, that, although the violence and fatality of the constitutional affection arising from phlebitis have been equally recognised, several explanations are given of the circumstances upon which these more immediately depend; and that, of the opinions expressed, even those which have most probability in their favor rest on somewhat uncertain grounds.

"The doubt, therefore, (says Mr. Arnott,) that still remains is rather attributable to the question not having received that careful consideration which is requisite in order to arrive at a satisfactory conclusion, than to any deficiency of materials to enable us to do so. In attempting this on the present occasion, I shall first offer a succinct account of several cases of phlebitis, where death was clearly referable to its occurrence, and the bodies were subsequently examined, so as fairly to represent the circumstances which characterised the origin, course, and termination of the constitutional affection; and, having done so, I shall proceed to draw such conclusions, accompanied by remarks, as the facts seem to justify." (P. 9.)

Several of the cases adduced were the result of Mr. Arnott's own observations; others are derived from various

* But, as the current of blood is centripetal in the veins, if any vitiated secretion enters them, the constitution is certainly more likely to suffer than if the same accident occurs in the arteries in which the course of the blood is eccentric.—Rev.

† Treatise on Gunshot Wounds, third edition, p. 299.

‡ Lectures on Surgery, by Tyrell, vol. iii. p. 205.

sources, but their selection has been restricted to cases of phlebitis arising from wounds, the injury being either slight, or, where extensive, the connexion between the inflammation of the vein as the primary affection, and the constitutional as the secondary, being too obvious to be mistaken. As the present investigation is strictly pathological, the details of treatment are omitted.

Having detailed seventeen cases of fatal phlebitis, Mr. Arnott enters upon the argumentative part of his paper.

"From these seventeen cases of fatal phlebitis, the first conclusion deducible is, the total disproval of the assertion that death results from the extension of the inflammation of the vein to the heart. In none of the ten instances following venesection was the superior cava affected, much less the heart; and in half this number inflammation had not reached to the subclavian, or even to the axillary vein. In the cases where the inferior cava had become inflamed, there is only one in which the heart is represented to have been actually implicated; and here, the deposition of lymph terminating at the entrance of the emulgent vein, the observation is, that 'there were marks of diffused inflammation extending to the right auricle of the heart, but the signs of adhesive inflammation terminated as described.'

"As a cause of death, then, the extension of the inflammation to the heart may be considered as a mere matter of assumption, the history of the error being simply this: Mr. Hunter's observations not having enabled him to form a decided opinion as to the cause of death, when this affection occurred in the jugular vein of horses, he suggested, as a query, whether it might not depend on the inflammation extending to the heart? By succeeding writers this suggestion was adopted without examination, and without any evidence being offered in its support. Indeed, the circumstance itself is of very unusual occurrence; for, with the exception of the instance just alluded to, I have only found two others in which it is alleged that the inflammation had extended from the vein to the heart, and in these the description is not very precise."

"As the preceding remarks have indicated, there are considerable differences in the extent of vein occupied by inflammation in fatal cases of phlebitis. Sometimes the disease has spread into several or most of the veins of a limb from that primarily affected; at others, it has not proceeded beyond the vessel in which it originally appeared. This last circumstance, with that of the fatal consequences sometimes ensuing from inflammation, limited to a few inches only of a vein, (as of about six of the cephalic, in the case of Arnold,) justifies the inference that the dangerous consequences from phlebitis bear no direct relation to the extent of the vein which is inflamed."* (P. 42.)

* M. Dance, who has lately detailed many cases of fatal phlebitis, comes to the same conclusion. It is his opinion that danger arises not so much from

In endeavouring to ascertain the nature of the connexion between the primary and secondary affections in this disease, the next question is,

"Whether the latter depends, as has been alleged, upon the entrance of pus into the circulation. We are thus led to inquire into the contents of the inflamed vessels, and on referring with this view to the cases which have been adduced, it will be found that in a number of them, where an open wound existed in the vein, pus was discharged from it during life. Whilst, in fourteen cases out of seventeen, pus, or pus in conjunction with lymph, was present in the vessel after death. In two instances no mention is made of pus, the contents of the veins being described in the one as 'adhesive matter;' in the other, where the cava was concerned, as 'flakes of lymph.' In one case only, where the inflammation occurred in a vein previously diseased, or in a vein the branches of which at least were varicose, neither pus nor lymph was found in the vessel.

"It results from this statement, that, although pus is present in the veins in the great majority of fatal cases of phlebitis, and that, although it should appear from the character of the general symptions, and the effects produced upon animals by the injection of a similar fluid into their vessels, that the passage of pus into the circulation is probably the principal, yet the circumstances do not justify us in regarding it as the sole cause of the secondary affection. In addition to the presumed absence of pus in two instances, and to its declared absence in a third, it may be remarked, that the early appearance of the symptoms in some cases seems scarcely to correspond with the time usually required for the production of pus, as in one which occurred to Mr. Freer, where they came on, suddenly, four hours after ligature of the saphena. If, then, the constitutional affection in phlebitis is to be explained by the introduction of a fluid into the circulation, which contaminates the blood and operates as a poison, this property must be attributed to inflammatory secretions generally from the vein, although not purulent; and it remains to be seen whether the symptoms of this affection are such as can be accounted for by the passage of pus only into the system." (P. 44.)

Before quitting the subject of the local affection, Mr.

the venous inflammation, as from the vitiation of the blood by pus having entered the general circulation. (*Archives Gen.* Fevrier, 1829, p. 182.) Blandin, also, (*Journ. Hebdomadaire*, No. 26, 1829, p. 591,) contends that the severity of the disease does not depend upon the extension of inflammation from the part of the vein originally affected, but that, on the contrary, it is the product of this inflammation, which mixes with and alters the state of the blood. M. B. endeavours to explain the cause of the different degrees of severity in the symptoms of phlebitis. Sometimes, he observes, the inflammation may affect only the external cellular sheath of the vein, or the internal coat of the vessel may be inflamed. In the former case, the constitution will not materially suffer; while, in the latter case, death is almost invariably the consequence.—Rev.

Arnott briefly alludes to a circumstance which has escaped the notice of those who have previously treated of inflammation of veins, viz. the point at which the inflammatory changes in the coats usually terminate.

"According to my observation, these changes are limited by the passage of a current of blood; where a trunk is concerned, the boundary being the entrance of a branch, and, where a branch is concerned, the boundary being the junction of this with the trunk."* (P. 46)

Mr. Arnott does not pretend to explain how it happens that this same inflammation, which has stopt short at the entrance or passage of a current of blood, may not only already have passed several currents, but have extended itself into the vessels conveying them.

The secondary affection in phlebitis usually shows itself in from two to ten or twelve days after the receipt of the injury which has occasioned the inflammation in the vein: when the vessel has been previously diseased, sometimes sooner. The symptoms are as follows:

"Great restlessness and anxiety, prostration of strength and depression of spirits, sense of weight at the precordia, frequent sighing or rather moaning, with paroxysms of oppressed and hurried breathing; the patient at the same time being unable to refer his sufferings to any specific source. The common symptoms of fever are present; the pulse is rapid, reaching sometimes to 130 or 140 in a minute, but is in other respects extremely variable. There is often sickness and violent vomiting, especially of bilious matter. Frequent and severe rigors almost invariably occur, sometimes to the number of three or four in the course of a few hours. The general irritability and deep anxiety of countenance increase, the manner is quick, and the look occasionally wild and distracted. When left to himself, the patient is apt to mutter incoherently, but, on being directly addressed, is found clear and collected. The features are pinched, and the skin of the whole body becomes of a sallow, or even yellow colour.†

"Under symptoms of increasing debility, and at a time when the local affection may appear to be in a great degree subsiding, secondary inflammations of violent character, and quickly terminating in effusion of pus or lymph, very frequently take place in situations remote from the original injury; the cellular substance, the joints, and the eye, have been affected; but it is more particu-

* The same inference would be drawn from the appearances found upon dissection of many of M. Dance's cases. (*Arch. Gen.* tome 18, 19.) M. Dance has not, however, adverted to this fact. Blandin states, that, "en général, le pus est circonscrit entre des valvules, au-dessus et au-dessous de veines collatérales volumineuses." (*Journ. Hebd.* No. 26, March 1829.)—Rev.

† This appearance of the surface is not invariably observed in cases of phlebitis.—Rev.

larly under a rapidly developed attack of inflammation of the viscera of the chest that the fatal issue usually occurs. Whether this is observed or not, death is always preceded by symptoms of extreme exhaustion, such as those of a rapid feeble pulse, dry, brown, or black tongue, teeth and lips covered with sordes, haggard countenance, low delirium, &c." (P. 52.)

The duration of this affection offers some variety. In some cases the patient dies a few days after the division of the vein; in others, the symptoms run on for several weeks.

The morbid appearances found in cases of phlebitis are very remarkable, and are such as we usually regard to be indicative of the recent existence of violent inflammation, and that, too, in various organs and distant parts of the body.

"In the chest, effusions of sero-purulent fluid into the cavities of the pleuræ and pericardium, exudation of coagulable lymph on the surfaces of the heart and lungs, hepatization of the latter organ, the infiltration of pus into its tissue, or small collections like a mixture of pus and lymph. Such appearances presented themselves in ten cases out of seventeen; in three, the thorax was not examined; in two, the condition of its contents is not noted; and in two, no diseased appearances were observed.

"In the cellular substance, intermuscular as well as subcutaneous, pus and sero-purulent fluid have been extensively deposited, sometimes in collections like abscesses, at others appearing more like an effusion into its cells than as resulting from the common process of inflammation. These collections most frequently occur in the vicinity of the joints. In two cases pus was deposited under the skin of the opposite forearm, near the wrist; in one, with inflammation of the knee-joint, into the intermuscular cellular substance of the corresponding thigh, and into that external to the joint of the opposite shoulder; in one, into the intermuscular cellular substance of the opposite leg and of both forearms; in one, into the interfibrillar cellular tissue of the corresponding pectoral muscle; and in another, between the sternal extremities of the two first ribs and pleura.

"A disease of the joints, consisting of a most violent inflammation of the synovial membrane, its distention with purulent matter, destruction of the cartilage, and baring of the bones. These changes, too, taking place in the brief space of a few days, the knee having been first attacked with pain four days before death, which, again, took place in sixteen from the date of the injury of the vein which caused its inflammation. In two other cases there was affection of the knee-joint, but the parts were not examined after death.

"In the eye, opacity of the cornea, injection of its blood-vessels, and destructive changes in its humors or coats, occurred in one case.

"Besides these affections, there were found in five instances, within the cranium, opacity and thickening of the tunica arachnoides, effusion between it and the pia mater, and increased secretion into the ventricles. In nine the head was not examined; in three no morbid appearances were noticed." (P. 54.)

The morbid appearances above described are met with singly or variously combined in particular cases; and, from the variety of situations in which these secondary local affections have been observed, it seems probable that there are few organs or parts in which they may not occur. "But, although some of them are generally found present, the constitutional affection in phlebitis occasionally proves fatal, without a secondary local affection having developed itself."

On considering the progress of the secondary affection in phlebitis, it is impossible not to be struck with the resemblance which this bears to that of diseases arising from the inoculation of a morbid poison.

"There is, in the first place, a local affection, (it may be of very trifling extent and severity,) upon which the secondary affection supervenes in the form of great constitutional disturbance followed by violent inflammations in one or more parts of the body. With this general resemblance to inoculated diseases, there is one to whose characters it more particularly approximates: viz. that which arises from the operation of poison received in wounds from dissection. We have an equally early appearance, and an equally rapid development, of symptoms nearly similar; succeeded by destructive inflammations in one or more situations remote from the primary affection, with equally fatal results. These secondary inflammations very much accord also in the parts which they attack. If the cellular substance is in a particular manner affected, in consequence of the introduction into the system of the poison of dead animal matter: this also occurs as a consequence of phlebitis, and some of the more obscure affections of the first class of cases may even, perhaps, be elucidated by the events of the latter." (P. 57.)

In a note Mr. Arnott observes, that the affection of the cellular substance, as a consequence of wounds received in dissection, is, like that from phlebitis, neither continuous nor limited to the side of the body on which the injury has been inflicted.

In corroboration of this statement, several cases are referred to.

"In conclusion, it results from the foregoing facts and arguments that death, in cases of phlebitis, does not take place from the inflammation extending to the heart; whilst the history and character of the symptoms which precede this event, the very small portion of vein which is sometimes found to have been inflamed, and the general presence of pus in its cavity, all tend to establish that the entrance of this fluid into the circulation is the principal cause of the alarming and fatal consequences of phlebitis; a similar influence being, perhaps, also possessed by any inflammatory secretion from the vein." (P. 61.)

*Part Second.*—The fact of purulent matter being sometimes deposited without much sign of previous inflammation, in a part of the body remote from one in a state of suppuration, has been long known in the history of medicine, under the name of abscess by metastasis; and it was formerly supposed that the matter was taken up and transferred, ready formed, to its new situation. Upon the subject of a translation of purulent matter, Mr. HUNTER's opinions were very decided: he discredited it as an occurrence, and as an operation he declared it to be absolutely impossible.*

" Mr. Guthrie,† in directing attention to a sudden and insidious attack of inflammation of particular parts, and especially of the lungs, as a cause of death after secondary amputation, (and of which inflammation, when the viscus affected is other than the lungs, he believes the termination is less rapid, suppuration taking place and abscesses being formed,) considered it as depending 'on the alteration which takes place in the sanguiferous system in consequence of the amputation, and the suppression of the discharge causing fever, and a determination to, and irritation in, a particular part.'‡—' The viscera in each person most predisposed to disease being most likely to be affected.'§ (P. 65.)

Pleurisy and tuberculous abscesses in different organs of the body, taking place subsequent to great surgical operations and suppurating wounds, have been the subject of several valuable communications from M. VELPEAU.‖ Mr. Arnott has not alluded to the theory which attributes the formation of these abscesses to a disturbance of the nervous system, the opinion itself being so purely conjectural, and the operation of the cause so undefined and unintelligible as to render this unnecessary.

" In fact, the only view of the subject, supported either by evidence or argument, is that which considers the origin of abscesses and inflammations in remote situations after injuries as connected with the absorption into the circulation of purulent matter from a wound. That they depend on the entrance of such fluid into the blood, the consequences which have been observed to follow phle-

* Hunter on the Blood, 4to. London, 1794; p. 360.

† On Gunshot Wounds, first edition, 1815; p. 73 et seq.

‡ Ibid. second edition, 1820; p. 235.

§ Ibid. p. 253.

‖ Revue Medicale, Juin, Juillet, et Decembre, 1826; Mai, 1827.

bitis simply sufficiently testify; and it becomes a question whether the occurrence of phlebitis, and the passage of pus from an inflamed vein into the circulation, is not of itself sufficient to account for the secondary affections of wounds, without its being necessary to resort to an absorption of the same fluid from their suppurating surfaces.'* (P. 67.)

The secondary affections succeeding to wounds, are effusions of pus and sero-purulent fluid into the cavity of the chest, and inflammation of the pleura; similar affections of the cellular substance; effusion of pus into, and inflammation of, the synovial membranes; depositions of pus and tuberculous abscesses in different organs of the body, viz. in the brain, lungs, heart, liver, spleen, and kidney.

"Now, when it is considered that abscesses have formed in various parts of the body from the ligature merely of a vein, as of the saphena;† that pus was deposited under the skin of the fore-arm in the case of Brancher; that rapidly destructive inflammation in the knee-joint took place in the case of Arnold; that the same occurred in the eye in the case of Dodging; that, where symptoms of inflammation of the chest were observed during life, the effects witnessed, on examination after death, were of very disproportionate degree and extent; and that effusions of coagulable lymph and sero-purulent fluid into the chest, together with abscesses in the lungs, were found where no symptoms had indicated their existence: I say, when it is considered that all these consequences ensued from so simple and definite an injury as the puncture, division, or ligature of a vein, it is impossible to resist the supposition that, where similar secondary affections have succeeded to a more extensive wound, they may in reality have originated in the same cause, viz. inflammation of a vein or veins.

"If such view of the subject is correct, we ought, on the one hand, in cases where the consequences already mentioned have succeeded to wounds and injuries, whether of the extremities or head, to find evidences of inflammation of the veins of the part which had been primarily or mechanically injured; and, on the other, to meet with similar secondary affections in cases where inflammation of the veins is known to be of common occurrence, as after parturition." (P. 68.)

That this is actually the case, Mr. Arnott proceeds to show. He relates four instances in which secondary affections of the viscera occurred after injuries of the extremities,

* Blandin (*Journ. Hebdom.* No. 26, March 1829, p. 585,) and Dance (*Arch. Générales*, Fev. 1829, p. 169,) without denying the absorbing power of the veins, are of opinion with Mr. Arnott, that the pus detected in the veins in cases of phlebitis is the product of inflammation of the coats of the vessel. By both these pathologists the question is ingeniously argued.—Rev.

† Carmichael, in Trans. of the King and Queen's College of Physicians, vol. ii. p. 346.

in connexion with inflammation of the veins in the wounded limb.

We now arrive at some interesting observations upon affections of the viscera, of the joints, and cellular substance, after injuries of the head.

Abscesses of the liver after injuries of the head long excited curiosity, and a variety of speculations were indulged in to account for the occurrence. Systematic writers afford us no satisfactory information as to the nature of those injuries of the head to which secondary affections of the viscera, of the abdomen and chest, succeed; neither do they explain the circumstances under which they occur. To supply this deficiency, and to enable us to form more satisfactory conclusions as to the origin and cause of secondary affections in distant parts after injuries of the head, Mr. Arnott refers to thirty-three cases, selected from various good authorities.

"From a consideration of these cases, it appears that affections occurring as secondary to injuries of the head were observed, in twenty-one, seated in the abdominal viscera; in five, in the thoracic; and in six, in the abdominal and thoracic conjointly. That they consisted of collections of pus in the liver and in the lungs; and of effusions of pus and sero-purulent fluid into the cavities of the chest. That, combined with some of these, there was further observed a deposition of purulent matter, in one case, in the substance of the heart; in one, in the kidney; in one, in the spleen; and in one, under the integuments of the back; in one, albuminous effusion on the surface of the intestines; and in one, inflammation of the liver, without the formation of matter. In two cases, inflammation of the surface of the liver, with suppuration, was the only morbid appearance observed. In one case, an affection of the joints, and the deposition of pus into the cellular substance around them, occurred without any disease of the abdominal or thoracic viscera having been noted." (P. 94.)

The injury which the head had sustained in these cases consisted, in twenty-three, of fracture or fissure of the cranium, in all compound, with the exception, perhaps, of two, where the circumstance is not stated. In ten, the osseous covering of the brain was neither fractured nor fissured, but there was always wound of the scalp. The morbid appearances within the cranium were various, and sometimes none were detected. In short, the only circumstance these thirty-three cases have in common is a wound of the soft parts.

"The general course of these cases seems to have been this, and in the great majority, twenty-four, it is so stated, that the patient for some time did well, having recovered his consciousness, where this had been lost, (which was frequently not the case,) was free from fever, and the wound suppurating kindly; that afterwards unfavorable symptoms took place, consisting of fever, rigors, nausea and vomiting, delirium, yellow colour of the skin, and sometimes, shortly before death, pain in the right hypochondrium, or affection of the chest. There was some difference in the period at which these symptoms appeared; but, of nineteen cases, the earliest of which was the seventh, and the latest the twenty-fourth day, the average was between the thirteenth and fourteenth day after the accident. The average period of the fatal termination of the same cases was between the twenty-second and twenty-third days; the earliest being on the fifteenth, and the latest on the thirty-seventh, subsequent to the injury. In one instance, not included in the above number, the patient did well until the eightieth day, when, on an attempt being made to remove a portion of bone adhering to the dura mater, general disturbance took place, and death in a few days. In another exception to the more ordinary periods, the same event occurred four months and a half after the receipt of the injury." (P. 96.)

In concluding this part of the subject, Mr. Arnott thus states his opinion :

"I think it will appear that abscesses and inflammations occurring in the viscera of the abdomen and chest, after injuries of the head, present a resemblance to similar affections succeeding to wounds of other parts of the body, sufficient to justify the inference that they arise from the same cause. That cause, I ventured to suggest, might be inflammation of the veins, the consequent production of pus in their cavities, and the entrance of this into the circulation; and, in accordance with this view, we find that, in the only cases in which the state of the part is described, (No. 21, from Schmucker, and No. 32, which occurred in St. Thomas's Hospital,) inflammation of the superior longitudinal sinus existed, its cavity, in both instances, containing purulent matter; in the one, with a firm, fibrinous coagulum, and in the other, with a layer of organised lymph on its inner surface. But we need not confine ourselves to inflammation of the sinuses within the cranium in cases of this description. It must be evident that this process taking place in the numerous veins which ramify between the two tables of the skull, and in those distributed to the soft parts externally, will be attended with similar consequences to those which succeed to phlebitis in other parts of the body." (P. 97.)

*Affections of the viscera, joints, eye, cellular substance, and skin, after labour.*—In examining the bodies of women who died after parturition, of (as it was supposed) inflammation of the uterus, various pathologists have stated that they found pus contained in the veins of that organ. It is but lately, however, that this subject has attracted especial attention; and certainly the facts which have been brought forward are highly interesting, and calculated to make us doubt the accuracy of opinions long entertained respecting the pathology of various maladies incidental to puerperal women.* Abundant evidence might be adduced to show that inflammation and suppuration of the veins of the uterus are followed by various secondary affections. Mr. Arnott offers sufficient proof that a violent and destructive disease of the joints takes place in the puerperal state. Dr. Merriman has seen such cases, none of which terminated favorably.† Upon one point we do not altogether agree with Mr. Arnott. He states, "Upon the connexion of this affection of the joints in puerperal women with inflammation of the veins, there is an appearance of direct evidence in two cases related by Velpeau, in his paper on Phlegmasia Alba Dolens, although the description is somewhat incomplete." M. Velpeau adduced these cases to show that *phlegmasia dolens* depends on inflammation of the veins. The disease which he describes has, however, little, if any, resemblance to phlegmasia dolens; and, even if it had, the cases he relates would not strengthen his doctrine; for of one he states that the internal tunics of the veins were healthy, and even pale; and again, "lex vaisseaux eux-mêmes n'offraient aucune alteration appreciable de leurs

* M. Dance relates eleven cases of inflammation of the veins of the uterus, with the appearances post mortem. In several of these instances there was extensive disorganization in remote parts of the body, such as the lungs and joints. (*Arch. Gen.* Dec. 1828, and Jan. Fev. 1829.) Dr. Robert Lee has detailed one case, and incidentally mentioned four others which occurred in his practice in September 1827, of inflammation of the spermatic veins and sinuses of the uterus. (*Med. Gazette*, Feb. 1829.) An interesting case is given in our Journal for May 1829, p. 420, from *La Lancette Française*, of inflammation of the womb, with considerable deposition of pus in the ovarian, right iliac and inferior cava veins. Blandin mentions a fatal case of inflammation of the veins of the uterus, after a ligature had been applied to a polypus of that organ. Baillie observes that "inflammation of the veins of the uterus often advances to suppuration, and the pus is generally found in the large veins of the womb. (*Morbid Anat.*, Wardrop's edit. vol. ii. p. 322.) Meckel, Ribes, Chaussier, and Schwilgué, have each published cases of inflammation of the uterine veins, and it appears probable that it is because the veins are inflamed, and contain pus, that puerperal fever, as it is called, is so frequently and quickly fatal.—Rev.

† After having related many interesting cases, M. Dance comes to the following conclusions upon this subject: 1st. That the joints of puerperal women are frequently attacked with inflammation, which quickly runs into suppuration. 2d. That such local affections ordinarily occur during the course of uterine phlebitis. 3d. That the progress of these affections of the joints differs from articular rheumatism, although some similarity may be observed between them. The attacks of the joints in puerperal women commences suddenly, and almost always quickly terminates by suppuration. One joint being affected, the inflammation does not suddenly attack other parts, as is frequently the case in rheumatism. (*Arch. Gén.* Dec. 1828, p. 491.)—Rev.

tissus." Neither in the second case have we any evidence of inflammation of the veins.*

The last circumstance to which Mr. Arnott alludes is the occurrence, in the puerperal state, of a disease of the eye, similar to that which took place in one of the cases he relates, from inflammation and suppuration in the vena saphena. Upon this subject an excellent paper has been published by Dr. Marshall Hall and Mr. Higginbottom, in vol. xiii. of the Transactions of the Med. Chir. Society. In all the cases, six in number, five of which came under their own observation, the affection of the eye took place in from five to eleven days after delivery. It was preceded and accompanied by serious indisposition, in every instance terminating fatally, and under symptoms of extreme exhaustion. Mr. Arnott has the merit of first elucidating the pathology of this curious affection.

In conclusion, Mr. Arnott believes, from the facts he has himself witnessed, and from evidence which he has collected from the best authorities, that the inflammations and abscesses of the extremities or of the head, or after the process of parturition, are attributable to the existence of phlebitis in the part of the body primarily affected.

"In concluding these remarks, the object of which has been to point out the relation between the primary and secondary affections in phlebitis, and to establish the introduction of pus, or other inflammatory secretion, from the surface of the vein into the circulation, as the cause of the latter, I have not felt myself called upon to advance any opinion as to the manner in which this cause operates in giving to some of the secondary affections their peculiar characters: I allude more particularly to the depositions of pus and lymph, unattended by those changes in the texture of the parts which usually precede the production of these fluids. I think it right, however, to state, that I must not be considered as regarding the matter so deposited to be actually that which has been brought into the circulation from the inflamed vein or veins. The disease of the eye, in which pus is not deposited, and the affection of the joints, exclusive of other considerations, clearly prove that the question is no longer one of a translation of matter merely, but one which involves the very difficult subject of the pathology of the blood; especially the share which diseased changes in this fluid have in the production of those phenomena which we are in the habit of comprehending under the term of inflammation." (P. 122.)

We have no doubt of the soundness of Mr. Arnott's doctrine, and those who are inclined to pursue the subject

* Recherches et Observation sur la Phlegmatia Alba Dolens. Par M. Velpeau. (*Archives Gén.* Octobre 1824.)—Rev.

will find it amply confirmed and illustrated by numerous cases, and apparently solid arguments, in various communications by Dance,* Bouilland,† Ribes,‡ Breschet,§ Blandin,|| and other French pathologists. To some of these writers, indeed, Mr. Arnott has referred.

We trust that at some future opportunity Mr. Arnott will complete the subject he has so ably commenced, by discussing the *treatment* required in the various species of phlebitis. We feel convinced that the more strictly the subject of venous inflammation, and *its consequences*, is examined, the greater reason shall we have for believing that many diseases, the nature of which is at present confessedly obscure, depend on a vitiated state of the blood, from the entrance of pus or other products of inflammation into the general circulation. The humoral doctrine is almost entirely exploded from the creed of modern pathologists; but surely Mr. Arnott has made out a case which must convince the most pertinacious solidist that he will sometimes find the first link of the morbid chain in a depraved state of the fluids.

We are much indebted to Mr. Arnott for this highly interesting work. Until the publication of it, the student would have sought in vain for any satisfactory exposition of the pathology of venous inflammation: for, although the subject has been incidentally touched upon by many writers, it has never been before considered with the ability and attention it merits.

---

*A Treatise on Syphilis; in which the History, Symptoms, and Method of Treating every form of that Disease, are fully considered.* By John Bacot, Surgeon to St. George's and St. James's Dispensary, and lately Surgeon to the Grenadier Regiment of Foot Guards.—8vo. pp. 280. Longman and Co. London, 1829.

So many and such conflicting doctrines have been broached within the last few years respecting both the nature and treatment of syphilis, and so confidently has each advocate maintained the solidity of his own views, and the efficacy of their application to practice, that they who have not opportunities of forming opinions for themselves must be much at a loss to determine upon what principles they are to act

* Archives Générales, Decembre 1818, and Jan. Fev. 1829.

† Revue Med. Avril et Juin, 1825.

‡ Idem, Juillet 1825.

§ Traité des Maladies des Artères et des Veines, tome ii.

|| Journal Hebdomadaire de Medicine, 28 Mars, 1829.

in the management of venereal complaints. One surgeon tells us that scruple or half-drachm doses of calomel are necessary; another assures us that an occasional grain or two of blue pill is amply sufficient; and a third rejects the use of mercury altogether, and is almost inclined, with Fallopius, to regard it as "omnium curationum acerbissima."

Notwithstanding, then, the labour and ability which have been expended upon this subject, it would be absolutely impossible for a student to come to any general and satisfactory conclusions by the assistance of any work which has been hitherto published in this country. There are, we grant, many valuable papers which might instruct him upon particular points of doctrine and practice; but a compendious and impartial view of the whole of the subject was yet to be desired.

We perused with much satisfaction and improvement the neat practical lectures of Mr. Bacot upon the subject of Syphilis, which were recently published in the "London Medical Gazette," and, as we anticipated, they have attracted sufficient attention to induce the author to present them to the profession in a more convenient form than that in which they originally appeared. The present volume is a reprint of these lectures, with many alterations and additions. It gives, in one view, the result of the opinions of most of the principal writers on syphilis, and will enable the reader to determine as to the justice of the peculiar views entertained by Mr. Carmichael and others, as well as of those opinions respecting the non-mercurial plan of treatment, advocated so freely in this country of late years.

The first chapters we shall pass rapidly over: they contain an amusing sketch of the history of the disease, from its earliest origin, and a survey of the fluctuating opinions that have, at different periods, each lived their little day, both in relation to the nature of the disease and the best mode of subduing its severity; together with an enumeration of various remedies, that at first were thought to possess every virtue, and now are known to be at best but useful auxiliaries. Many of them, indeed, are now altogether expelled from our therapeutic catalogue.

Mr. Bacot very judiciously ushers in his work by the undeniable truth, "that never, since syphilis became an object of professional inquiry, has there been a period when some positive and determinate doctrines were more imperatively called for than the time in which we live." Let us turn to the recorded opinions of modern writers: what do we find? By one we are taught that there are three or four venereal diseases; by another, that scarcely any thing but pseudo-syphilis is now to be met with; by a third, that there neither is nor ever has been such a disease in existence. We have already shown that the same discrepancy exists as to the treatment of the disease.

Mr. Bacot does not consider the name by which a disease is designated as a matter of much importance. Nor is it, provided always we are agreed as to the precise value and signification of the term employed. The term syphilis is here generally employed to denote the primary affection; the constitutional symptoms are included under the denomination of secondary syphilis. "For the sake of varying the expression," Mr. Bacot occasionally speaks of the "venereal disease, or lues venerea: still this latter phrase appears to be too vague and general." We may just observe, that the euphony of a sentence is too dearly purchased by the employment of a confessedly indefinite expression.

Having brought down his historical sketch of the disease to the writers of the middle ages, the author trusts he has convinced the sceptical that there is really no well-founded reason for believing that any disease generally affecting the constitution, or tending to the destruction of the patient, was known to the Greeks, Romans, or Arabians, as the direct consequence of connexion between the sexes. In our opinion he satisfactorily shows that the origin of syphilis must be referred to the latter years of the fifteenth century, and that there are sound reasons for doubting the commonly received opinion of its American origin. Excepting in the rapidity of the march of the disease, the principal features were the same in the early part of the sixteenth century as at this time. They are mitigated in severity, but in kind they remain unchanged.

Wiseman was the first author who observes, from his own experience, that it often happens some men will be infected, whilst others shall escape with impunity, from the embraces of the same woman. "I have known," he says, "twenty men lie with one and the same woman the same day, and only one of them affected, though the rest equally deserved it." This very sensible practitioner was also particular in directing venesection before the commencement of the mercurial treatment. He believed that by this means, assisted by purging, the remedy is better borne and more efficacious.

The absurd notions which existed, and the dangerous practice which was consequently adopted, towards the close of the seventeenth century, render it easy to imagine the number of victims such treatment must have produced; "and we may very well comprehend the horror with which the pox was regarded in those days, and why it was made use of as one of the bitterest imprecations; since it would appear to have been almost impossible to escape either mutilation or death from the disease or the remedy." At this period there were, however, some practitioners who entertained opinions relative to syphilis more in conformity with the views which have lately caused so much discussion in this country.

"Of these, David Abercrombie is the most remarkable: he published a short dissertation on syphilis in 1684, in which he condemns mercury entirely, and declares that the vegetable remedies are alone sufficient to effect the cure of nearly every form of the disease, though he admitted the necessity of occasionally employing mercurial pills: but later in life he seems to have changed, or at least modified, his opinions very much, and contents himself with recommending the substitution of the mercurius dulcis for the mercurial inunction, and restricts his censures of the mineral remedy to the condemnation of salivation in patients of certain habits and constitutions." (P. 37.)

In the early part of the eighteenth century, this milder method of giving mercury obtained many advocates. Warm discussions, of course, arose between these practitioners and the favorers of the older doctrines. BOERHAAVE was greatly influential in bringing the profession to a more just and temperate appreciation of the powers of mercury. He placed much confidence in the decoctions of sarsaparilla and guaiacum. VAN SWIETEN, MORGAGNI, and others, entertained similar opinions. The latter eminent authority indeed observes, "When I went to Bologna, as a young man, both the external and internal use of mercury was nearly deserted, and I never heard of its being used during the eight years I remained there, either one way or other, in the treatment of the venereal disease." Still there are repeated proofs that, in the first half of this century, the practice in this disease was very far from settled. Sir WM. FORDYCE advanced very closely to the mode of practice advocated and employed by many surgeons of the present day; but yet at that period his experiments made but little impression upon medical men in general, "for we are told by Mr. Bromfield, almost at this very time, that he never saw a single instance in which the sarsaparilla cured the venereal disease without the assistance of mercury, either given with it or taken previously; and Mr. Pearson remarks that his own observations coincide entirely with those of his predecessor."

From the not unfrequent obstinacy of the disease, however, even when mercury was employed, and from the evils that this remedy itself produced, other curative agents were still sought after. Opium, cicuta, and the nitrous acid, may be especially named. The first and second appear to have only a palliative power, but the nitrous acid has a stronger claim upon our attention.

"One of the reasons that contributed to support the reputation of this remedy, was the obvious effect it had in producing inflammation and swelling of the gums, and, as mercury possessed a similar power, many theorists imagined that the medicinal effects of both remedies were the same; and hence arose the hypothesis that mercury owed its curative powers to the oxygen contained in the majority of its preparations." (P. 40.)

In spite of all the efforts made by surgeons, at various periods, to supersede the employment of mercury, its supremacy was thoroughly established at the close of the eighteenth century.

"It was given indiscriminately for every breach of surface on the genitals; scarcely could any cutaneous affection escape the suspicion of a syphilitic origin; nocturnal pains were generally condemned to inunction, without mercy or discrimination; and the state of the venereal wards of our public hospitals will not easily be forgotten by those who are old enough to have witnessed the disgusting details they afforded: nay, I am sorry to observe that this evil has scarcely been abolished entirely in our own days." (P. 41.)

The labours of Mr. Hunter obviously led the way to much that has been more fully developed by others: his researches into the nature of the venereal poison, his original notion of certain affections resembling syphilis, as well as numerous other novel and ingenious ideas scattered throughout his work, evince the original and comprehensive mind of that great man. Mr. Bacot is, however, compelled to admit that John Hunter's work has many and serious faults.

"There is, however, yet another writer whose labours demand a little of our notice, though (by a fatality which is often observed, and not to be accounted for,) his work made but little impression on the public mind, and seems now to be almost forgotten: I allude to Dr. Clutterbuck's pamphlet, published in 1799, and entitled 'Remarks upon some of Mr. J. Hunter's opinions on the Venereal Disease.' The most remarkable passages of this work relate to the belief of the possibility of curing many forms of the venereal disease, not only without mercury, but without medicine of any kind; or, in plain language, admitting that they might undergo a spontaneous cure. Thus it may be seen how very nearly this gentleman advanced to the conclusions which have since been the result of direct experiment; and that, in fact, as a late excellent writer has remarked, he may justly claim the merit of having distinctly pointed out to us that the mere circumstance of a disease giving way and being cured without mercury, is no proof that the case is not venereal." (P. 44.)

The third and fourth chapters are principally devoted to an examination of the writings, and a detail of the opinions, of authors of the present day. The Peninsular war opened to the medical officers of the British army new views relating to syphilis, and they quickly communicated the information they acquired. Mr. Ferguson first published the results of his experience in Portugal. It appears evident that this gentleman considered the conclusions to which he arrived as totally inapplicable to this country, though true as far as they regarded the natives and climate of Portugal. The facts we obtain from Mr. Ferguson are principally the following:

"It was customary among the native practitioners in Portugal to cure all primary venereal affections with topical applications only; the native soldiers, as well as those in civil life, were accustomed to perform their duty, and follow their usual avocations, with sores on the penis, not merely such as were of a trivial nature, but such as made Mr. Ferguson shudder to look upon; the only difference in the treatment adopted by the military and civil practitioner in such cases being, that the latter generally combined the decoction of the woods with the local remedies; but in both instances the use of mercury was reserved for those in whom the bones had become affected, when a very small quantity, usually of calomel, was prescribed, together with Dover's powders, warm baths, and other sudorifics. Dreadful examples of mutilation did, indeed, sometimes occur; but these bore no proportion to the number of those who had suffered from the primary symptoms of the disease; and the affections of the bones, when they did occur, were usually slight; thus proving that, in this climate at least, the complaint had become so much mitigated as to run generally a mild course, until it at length exhausted itself spontaneously.

"Very different, however, was the progress of the symptoms in the British army: among the soldiers its ravages were so frightful, that Mr. Ferguson says it is probable that more men had sustained from this cause the most dreadful of all mutilations, during the four years the army had been in Portugal, than the registers of all the hospitals in England could have produced in the last century; so that not only were the primary sores more intractable to mercury than in England, but also secondary symptoms made their appearance in no small proportion, even whilst the constitution was actually under the influence of mercury." (P. 46.)

Mr. Rose next gave his assistance to the elucidation of this interesting subject. The results of his experiments in the hospital of the Coldstream Guards, during a period of nearly two years, were published in 1817. In this publication Mr. Rose announced that he had cured *all ulcers* on the parts of generation, as well as the constitutional symptoms to which they gave rise, *without* mercury. Some of the cases might not have been syphilitic, but the majority of them were so without any doubt.

Mr. Guthrie also confirmed the statement that *all ulcers* indiscriminately may be cured without mercury. He observes, however, that, in many of these cases, the cure was very tedious, and the cicatrices of the sores were frequently giving way. The secondary symptoms were mild, but tedious to cure. Mr. Guthrie contrasts the result of his practice with mercury whilst surgeon to the 29th regiment, between 1801 and 1809; and he remarks that, during this period, when his patients generally underwent a moderate course of mercury, he very seldom had a case of secondary syphilis. From the non-mercurial treatment, the average number of cases of secondary symptoms was at first stated to be one in ten, but Mr. Bacot believes that one in four or five would have been more correct.

From the statements of the writers above named, and many others referred to by the author, it appears to be fairly deducible,

"1st. That all sores of the genitals, without exception, are curable without mercury. 2dly. That secondary symptoms occur in the proportion of at least one in ten of those cases where no mercury is used; whilst, on the contrary, the proportion of such cases is only as one to seventy-five where that remedy has been employed. 3dly. The possibility of curing nearly all the forms of the secondary syphilitic symptoms without the assistance of a particle of mercury. 4thly. The mildness of these symptoms, which, excepting in about half a dozen instances, were confined to eruptions in the skin and ulcers in the throat. 5thly. That the period required for the cure of the primary sores by the non-mercurial plan was not in general greater than where mercury was employed; though it is admitted that the cicatrices of the sores remain frequently in a state of disease, often ulcerating again, and that the secondary symptoms, though not violent, were very tedious, and, when apparently cured, would not unfrequently recur again and again." (P. 55.)

These are doubtless very important conclusions, but they are modified by the following sensible remarks of Mr. Bacot.

"The experience of a few more years, whilst it has left the facts above cited untouched and uncontradicted, has amply shown that the proportion of secondary symptoms, as well as their obstinacy, the slowness and uncertainty with which primary ulcers heal, and their frequently breaking out again under the non-mercurial system, rendered it highly inexpedient, and in fact impossible, to introduce this practice into general use: nay, more, in several instances, even among the military, little accustomed to regard consequences, it began to excite uneasiness; the proportion of cutaneous affections, of ulcered throats, of pains in the larger joints, and other concomitant symptoms, became a serious evil, and induced many regimental surgeons to remodel their practice, and to adopt a plan of treatment less exclusive with regard to mercury.' (P. 56.)

The facts proved by the non-mercurial treatment are, however, valuable in many respects. They show that we may safely and advantageously dispense with the use of mercury, where it disagrees with the constitution. If fever arises, or local or general pains are induced, we may patiently wait for a more favorable moment for exhibiting the medicine. We may apply to ulcers on the genitals the same principles of cure which would apply to sores on any other part of the body. If the patient is prone to struma, we may forbear its use.

"Or, when necessary to do so, we may prescribe it either in so mitigated a form, or under such combinations, as to disarm it from all those dangers which occasionally render its exhibition a cause of more real suffering than the disease itself; and yet let me not have it imagined that I am one of those who recommend the exclusion of mercury from practice in the venereal disease: on the contrary, it is my object to prove that in the vast majority of cases it is our sheet-anchor." (P. 60.)

From the time of Mr. Hunter's publication, a new page of our history may be said to be opened. Until then syphilis was not doubted to be one disease, and all the variety of symptoms were attributed to one poison; but from that date a new host of diseases were admitted into the catalogue of human woes. These were said to resemble lues in appearance and progress, but yet they were thought not to be syphilitic. To this subject Mr. Bacot next turns his attention. It is, in truth, the foundation upon which Mr. Carmichael has built his theory of a variety of syphilitic poisons. It must be observed, that much of the reasoning employed by Mr. Hunter, and subsequently by Mr. Abernethy, has now fallen to the ground, since the fact of all forms of primary ulceration being curable without mercury has been admitted; for all their distinctions rest upon the converse of that proposition.

Mr. Bacot speaks in high terms of Mr. Evans' work on Ulceration of the Genitals. He has no hesitation in saying that Mr. E. has done more than any other writer towards discriminating those sores which are the produce of impure connexion, from those which are not so. Mr. Evans is, however, an advocate for more than one kind of venereal disease. In the very outset, indeed, he draws the same distinction as Hunter and Abernethy had previously done; a distinction which does not in reality exist. All that Mr. Evans enables us to assume is, that *it is possible* to ascertain clearly and distinctly certain forms of ulceration, which are not the produce of impure connexion; and, moreover, his observations go far to show that sores, not being so produced, are not followed by constitutional affections.

Mr. Bacot examines the opinions of Mr. Carmichael with much attention, and enters his protest against their accuracy; for he cannot acknowledge more than one venereal disease. Mr. C., on the contrary, professes to have traced several distinct morbific poisons to their source, and to point out also the consecutive symptoms belonging to each. Without presuming to settle such a question by the intervention of our dictum, we must be allowed to say that we agree perfectly with Mr. Bacot upon this point. The doctrines of Mr. Carmichael are plausible and ingeniously put, but, in our judgment, they do not stand the critical examination to which Mr. Bacot has submitted them. From the experience of Hennen, Rose, Guthrie, &c., as well as from his own observation, it appears to the author that it is not possible, at present, to form more than one or two general conclusions, "the principal of which appear to be, that the papular is by far the most common form of all the eruptions met with in syphilis; that the sloughing sore, in its most acute form especially, is often unattended by any form of secondary symptom; and that the tubercular eruptions are very often the result of the inadequate exhibition of mercury."

"I am led to conclude that, in the present state of our knowledge, we cannot give any satisfactory answer to my third query: that is to say, we are not able to trace, with certainty or regularity, distinct forms of constitutional affection to distinctly marked forms of primary ulceration; and this leads me to believe that there is one venereal poison only, and that the variations we observe in the symptoms, both locally and generally, arise from difference of habit, difference of treatment, perhaps from different stages and conditions of the virus itself, and from many minute and undefined circumstances with which we are at present unacquainted. Moreover, it is equally clear to me that, if we are to restrict the employment of mercury to that sore in which all the characters of the Hunterian chancre are united, we should have very few opportunities of employing it at all, but should continue to be disgraced by a succession of secondary symptoms, which the more frequent and judicious employment of that medicine would most assuredly prevent." (P. 79.)

In the ensuing chapter the nature and effects of the syphilitic poison are considered. The poison of syphilis is one, *sui generis*, affecting the human race only, and subject to laws differing materially in many respects from those which regulate other morbific poisons; among which differences, that of being communicated an indefinite number of times is not the least considerable. Mr. Hunter justly remarked, that we know nothing of the nature of the venereal virus. Its effects we are too well acquainted with. There are some respectable authorities who believe that the constitution may become affected without any previous breach of surface, from a bubo solely, or by the communication of the secondary symptoms from one person to another, merely by their sleeping together in the same bed. Mr. Bacot relies but little upon such statements: he is inclined to believe that some deception may have misled the surgeon, to which in such cases there are often such obvious and powerful motives.

He is of opinion that, although the doctrine of the identity of gonorrhœa and syphilis is not to be acceded to in its fullest extent, those who have supported it have more reason upon their side than might be at first supposed.

"Thus I am inclined to believe that, although the vast majority of cases of discharge from the urethra, attended with pain in making water, which are the consequence of sexual intercourse, and have therefore the common name of gonorrhœa applied to them, are, in truth, merely local affections of different degrees of intensity and duration, depending much upon the peculiar temperament of the person affected by it, and other accidental causes; yet still I acknowledge the existence of a species of gonorrhœa to which the term of venereal, or syphilitic, has been applied; and I further believe that this species may lead to ulcerations of the throat and palate, to ophthalmia, to eruptions, to swellings and pains in the joints, and, finally, to affections of the periosteum and bones." (P. 90.)

Mr. Bacot takes a brief survey of the opinions of various authors upon this subject. The following remark, he observes, which has been made by Mr. Sawrey, cannot be well replied to: "If in any *one instance* the inoculation of gonorrhœal matter has produced chancre, there is an end to the question." Swediaur declares that he has seen several examples of ulcers in the throat, and other evident symptoms of lues, appearing in consequence of a blenorrhagia, (i. e. gonorrhœa,) without there having been the least appearance of chancres either on the thighs or genital parts. Mr. Bacot has himself seen more than one unequivocal example of ulcers in the throat, eruptions on the skin, ophthalmia, and even affections of the periosteum, following gonorrhœa only. He also states he knew many cases where patients affected with blenorrhagia, without any ulcer, communicated chancres, and reciprocally. A prostitute sometimes gives one man a clap, another chancres, and a third both at once.

"Thus far Dr. Swediaur's facts go to establish this position, and he next endeavours to explain why it is that mercury is not necessary for the cure of a clap, and how it happens that secondary symptoms so seldom follow this form of the complaint: this he believes to be owing to the structure of the parts, as well as to the internal surface of the canal being defended by a quantity of mucus, by which the virus is much diluted, the sides of the urethra are defended, and consequently the formation of an ulcer prevented. He might also have added, that nine cases out of ten of discharge from the urethra are not really cases of syphilitic gonorrhœa." (P. 97.)

Vigarous and Lagneau, men of extensive experience and careful observation, confirm the above arguments. To rebut this evidence, we have only the experiments of Mr. Bell; and Mr. Bacot is, we think, justified in asserting that, if they were twice as numerous, they would not be sufficient to overthrow the positive testimony of so many writers of credit and authority.

It is of evident importance that the surgeon should bear in mind that many, or perhaps all, the symptoms of gonorrhœa may arise from other causes than impure connexion. Female children are often affected with a severe discharge from the pudenda, which is very obstinate. We have seen many examples of this kind.

"In the instance of adults of either sex, it is, however, obviously impossible in every case, or indeed in most cases, to form an opinion as to what discharges may be followed by after consequences, or to distinguish them from those that will not: the mere intensity of the symptoms is not always a safe criterion to judge by. All, then, that we are enabled with certainty to say is this, that it is possible to pronounce on many occasions that a gonorrhœa is not venereal: thus, for example, if a discharge came on a few hours only after connexion, if it has continued several days without inflammatory symptoms, if the patient has been liable to some discharge after any excess either of venery or of wine, in all such cases the probability is that the patient labours under some other diseased condition of the urethra, and that, though the intercourse of the sexes may have been the exciting cause, still there may be no imputation upon the cleanliness of the female; and the same rules will apply with still greater force to females, who are so subject to discharges from many causes unconnected with the commerce of the sexes." (P. 101.)

The symptoms of gonorrhœa are accurately described, as they occur in the male and female.

"The cure of gonorrhœa is to be undertaken upon the principle of subduing severe inflammatory action, reference being had to the nature and functions of the part affected. If the patient, as sometimes happens, suspects the probability of his having received the infection, and carefully watches his feelings, he is often enabled to notice the uneasy sensation at the orifice of the urethra, and a slight turgescence of the lips, for some hours before any increased secretion is discovered; sometimes even for a whole day previously. Should he in this condition apply for relief, it is more than probable that the use of an astringent injection, so as to produce some irritation of the internal membrane, will altogether supersede the disease. This I have effected in numerous instances, and with one precaution the practice is as safe as it is often efficacious. In those cases of incipient discharge, where there is no pain or scalding in making water, this plan may also be had recourse to, with the almost certain effect of suppressing the discharge often in forty-eight hours, or even less; but it must be remembered that, in either case, if the employment of the injection the first or second time produces great pain, and that the heat in making water is rendered much more severe, the injection must be directly abandoned." (P 111.)

The injections considered most effectual for the purpose above mentioned are either two grains of sulphate of zinc to an ounce of water, or about a grain to the ounce of the sulphate of copper. Mr. Bacot speaks very cautiously respecting the practice recommended by some surgeons of employing a strong solution of lunar caustic as an injection. In irritable habits it will act with great violence, and produce much mischief. We have a friend at this moment confined to his bed with violent and even hazardous inflammation of the penis, brought on by this improper and unjustifiable treatment.

The personal experience of Mr. Bacot does not enable him to commend the copaiba as a remedy in the first few days of a virulent gonorrhœa. The cubebs he does not estimate above other plans of treatment. In aggravated gonorrhœa, rest is considered absolutely necessary. We are confident that a want of attention, or rather the utter neglect of this precaution, in ordinary practice, is the cause of most of the severe and intractable symptoms which so frequently distress the patient and perplex the surgeon. Local bleeding is frequently necessary. Cupping from the perineum is preferred to leeches; the latter often produce irritation of the skin, œdema of the prepuce, and phymosis. Free purging in gonorrhœa is improper. When the most urgent symptoms are allayed, the author then employs copaiba or cubebs.

Untoward circumstances occasionally arise. For example, the scalding sometimes continues when every other symptom has given way. "I have seen this amount to a very serious evil, and have frequently witnessed the difficulty attending its removal. I think the use of muriatic acid, four drops to four ounces of water, used as an injection, has succeeded better than any other remedy, either external or internal, that I am acquainted with, in controlling this symptom."

When, after the employment of free bleeding, the inflammatory symptoms of swelled testicle still continue to linger, the tartar emetic will be found an excellent remedy. "The mode of administering the tartar emetic is in a mixture containing two, three, or four grains dissolved, with the addition of an ounce of Epsom salts, in six or eight ounces of water: of this mixture the patient is directed to take two or three tablespoonsful every half hour, or oftener, until vomiting is excited; after which the dose is repeated at intervals of three, four, or six hours, according to circumstances."

In mild cases the vinum colchici, in small doses, has been found serviceable. Many surgeons object to the employment of injections, and condemn them as productive of stricture and other diseased conditions of the urethra; "but these are groundless apprehensions."

We are compelled to pass over many other good practical remarks upon the primary and remote consequences of gonorrhœa.

In the next chapter the primary symptoms of syphilis are considered. The term chancre is now almost discarded from our vocabulary: we are contented to call the primary affections on the parts of generation by the name of ulcerations, adopting a distinctive epithet to them, such as is afforded either by their appearance or situation. To this change Mr. Bacot does not object. The word chancre is unscientific in its origin, and useless in its application: it implies, in fact, a cancerous sore. No definite description can include all the forms and species of syphilitic ulcerations. With the exception of one circumstance only, an inaptitude to heal, they may present every variety of appearance which a breach of surface may be supposed to assume. "Generally speaking, there will be a considerable difference in the activity of the poison, according as it has been applied to the cutis, to the cuticle, or to the glans itself. Ulceration will take place earlier in the latter situation, and latest of all on the skin of the penis." In some cases, the poison appears to have been inactive for three or four weeks. Mr. Hunter relates two instances of a still more tardy infection. In one case, seven weeks elapsed before the chancre made its appearance; in the other, two months. Most commonly it begins to exert its power within a week or ten days after connexion. The first appearance of a syphilitic ulcer, according to the united testimony of all writers, both ancient and modern, is in the form of a pimple or small pustule, whenever it has been traced to its commencement. This fact Mr. Bacot urges as a strong argument in favor of the unity of the syphilitic poison.

"It may be generally admitted that hardness is an accompanying mark of all syphilitic sores; that they also usually put on a figure approaching to the circular, and are not necessarily attended with much surrounding inflammation and pain, though they are liable to be attacked by it, and then their sensibility becomes greatly augmented: but there are also a few diseased appearances to which the parts of generation are liable, which it may be as well to endeavour to distinguish from the different forms of syphilitic ulcerations. These are chiefly excoriations, herpes either of the internal prepuce or of the cutis itself, common phlegmonous boils, or small apthous-looking ulcerations occurring in clusters; and most of these may come on independently of sexual intercourse, though it is obvious that few men are able positively to assert that this is the case; and hence arises the alarm which any breach of surface on these parts immediately occasions. Excoriations most frequently take place in those persons who have the prepuce long, and where cleanliness is not strictly observed, and the natural discharge from the parts is in great quantity." (P. 151.)

Some men rarely have connexion without its producing a slight breach of surface; but the characters of these excoriations will not be mistaken for syphilis.

"Another description of sore is sometimes met with more particularly round the corona glandis; that is, very minute apthouslooking points, which are sometimes in clusters, and at others extend around the whole of the glans; some will heal whilst fresh ones break out. They are totally devoid of pain, and are best got rid of by the application of the lunar caustic, or a wash composed of the acetate or sulphate of copper in proper proportions. I have known these appearances last a considerable time, but they are not certainly followed by any constitutional affection, and may be trusted entirely to local applications of the stimulating kind." (P. 153.)

The appearances of the gangrenous, sloughing, and apthous ulcers, and of the raised ulcer of the prepuce, are minutely described, and their appropriate treatment pointed out.

"In the administration of mercury, one precaution is absolutely necessary: either perfect confinement to the house, or such care in the exposure to weather, and such precautions against damp clothes or wet feet, or other sudden transitions of temperature, as shall be equivalent to it. These are old precautions, it is true; so old, that I am sorry to say they are worn out; and now we meet with people every day who are getting cured of syphilitic complaints, and taking a mercurial pill or two, pursuing their usual business, or (what is still worse) usual debaucheries, without fear of the consequences; but this is a line of conduct which I never would sanction." (P. 161.)

In many of those cases of sloughing sores in which there is no time to wait for the effects of mercury given in the usual way, the mercurial fumigation baths are recommended. Mr. Bacot is inclined to think that the operation proposed for phymosis is seldom necessary, and he would restrict it to those cases wherein the pain and tumefaction are very great, and the ulcerations within are of that highly painful and inflammatory character leading to the gangrenous sore.

The next chapter describes the mode in which buboes occur, their varieties, and treatment. One caution Mr. Bacot particularly inculcates, "never prescribe for a bubo as a solitary symptom, whatever rank of life the patient may be in, without examining the penis, if a male, or the pudenda in the female, if you can possibly avoid it; for patients, under these circumstances, will occasionally deceive if they can, even though it be at the expense of their own health."

Another local affection, if not exactly among primary ones, at least the consequence of them, is the formation of warts.

"They may be snipped off with scissors, or tied with a fine silk; they may be taken off by caustic, or destroyed by the powder of savine, by the liq. plumbi acetatis undiluted, or by the tinctura ferri muriatis, by a strong solution of oxymuriate of mercury, or by the strong acetic acid; but the extent, description, and mode of attachment of these excrescences should be our guide in prefering one or other of these remedies: for example, if the wart is large, with a small neck, I should cut it off with the scissors, and touch the cut surface with the lunar caustic. This will have a double effect: it will stop the bleeding, and prevent the growth of the wart, for they are all inclined to sprout again. If the mass was of the fungoid kind, I should, on the contrary, dip a piece of lint in the tinct. ferri muriatis, and lay it upon the part, or sprinkle it with the powder of savine; or if it was circumscribed, but still of a soft nature, perhaps I should prefer including it within a ligature; but, whenever the surface from which the warts have grown is extensive, it will be necessary to wash the part for some considerable time afterwards with a strong solution of the sulphate of alumine or zinc, or of the oxymuriate of mercury, which will tend to prevent their reappearance." (P. 193.)

Chap. X. *Constitutional or secondary Syphilis.*—This train, or succession, of symptoms is the consequence of the absorption of the poison, from some breach of surface on the body, or occasionally in consequence of a virulent gonorrhœa. Some very recent writers teach us that, though very rarely, yet still there are cases in which the constitution has been affected without any primary symptom having preceded. Upon this point Mr. Bacot is sceptical; and we agree with him in doubting the existence of any such cases, although a positive denial of such exceptions to the general fact would not be justifiable.

The order in which the various parts of the system are usually attacked by secondary symptoms, is next remarked upon.

"It was, however, formerly thought that no deviation from this regular succession ever took place, but that the progress of the symptoms was uniformly from bad to worse, until the constitution was fairly worn down by the disease; but we are now, on the contrary, convinced, from ample experience, that this is not even commonly the case: that nodes and caries of the bones are comparatively rare; that syphilis can, and does most commonly, exhaust itself upon the first order of parts; and that those terrible cases of diseased bone are more rationally attributable either to the action of the disease on a strumous habit, but more especially to the rash and ill-advised employment of mercury in certain peculiar constitutions." (P. 198.)

The time which usually elapses before the appearance of secondary symptoms, is the next subject of consideration. It is certain that, within six weeks after the occurrence of the primary sores, or even before they are healed, the patient's health will often begin to fail. Nocturnal pains come on, and an eruption quickly follows; but this Mr. Bacot believes chiefly takes place where no mercury has been exhibited. If that remedy has been employed inadequately, a longer space of time will usually elapse, and from four to six months is by far the most general period. In some cases the action of the virus may be suspended for some time. It must not be forgotten that there are but few of the symptoms of secondary syphilis so peculiarly and exclusively belonging to that disease, that we are at once enabled positively to assert the real nature of every case that comes before us. The abuse of mercury produces many affections that simulate closely those of the venereal disease, and peculiarity of constitution affords many other striking resemblances. To form a correct opinion, then, we must carefully attend to the history of each case.

"A syphilitic sore throat has been described as one unvarying symptom, but it has several varieties; and these varieties, as well as those of the primary sore, and the particular character of bubo, require a modification in practice. It requires also to be distinguished from the sore throat induced by mercury, for which it may be easily mistaken; and this would be a most unfortunate error." (P. 200.)

Of eruptions there are many distinct characters, as the papular, the pustular, the tubercular, which may be considered as the heads of so many distinct classes of eruption. Thus far Mr. Bacot's observations coincide with those of Mr. Carmichael; though he "utterly denies the regular conjunction of either of these peculiar forms of eruption with particular species of ulceration." Mr. B. is moreover "decidedly convinced that the occurrence of any or all of these secondary symptoms may be prevented, in the great majority of instances, by a judiciously managed mercurial course." The expression employed in this sentence, it is true, admits of exceptions to the doctrine inculcated, but still we think the argument is too strongly put: we cannot admit that "the appearance of secondary symptoms is, in most instances, a stain upon the reputation of the practitioner who has had to treat the primary affection," although we are perfectly aware of the occasional ill consequences resulting from the ignorance or negligence of the surgeon who directs the treatment of the disease in its first stages. Have there been, or are there, any surgeons, whatever may be their ability or their mode of treatment, who have not more frequently to contend with secondary symptoms, in cases which they have themselves treated from the commencement, than would be supposed from the manner in which our author has expressed his opinions upon this subject?

The directions given by Mr. Bacot to ensure the efficacy of the mercurial course are highly judicious.

The secondary symptoms are next described seriatim, and a few pages are devoted to the subject of syphilis as it affects pregnant women and infants. That infants are occasionally born with symptoms of syphilis, of too undoubted and well-marked a character to admit of dispute, cannot, we think, be denied by any practitioner of ordinary experience. We confess, however, we have not detected "a peculiar shrill tone of voice" as one of the symptoms of this disease in infants.

The following case is highly interesting:

"A young gentleman, just before he married, had been attended for venereal complaints. Thinking himself safe, he married a beautiful woman, who was delivered of a fine healthy boy at the end of ten months. During her second pregnancy, the husband declined visibly in his health, and, within five months of the second delivery, he had venereal ophthalmia, and a suspicious fungous excrescence round the anus. At the time that the lady was confined, in addition to the above symptoms, the husband had an ulcer at the back of the palate, extending towards the larynx. He then submitted to a course of mercury, and got finally well. During the time that the gentleman was under treatment, the infant's condition excited attention: it was squalid, and full of eruptions scattered from head to foot, and appeared to swallow with difficulty. It could not suck, and was fed upon goat's milk. On the mother not the smallest mark of venereal infection was to be found. The child was cured by rubbing ten grains of the stronger mercurial ointment into the soles of the feet, and continuing this treatment until the symptoms all yielded." (P. 253.)

The concluding sections are occupied by remarks on mercury and its various preparations, and upon the various derangements of health which the use of this medicine so frequently produces. Mr. Bacot joins Mr. Abernethy in extolling the advantages of mercurial fumigations. He believes with him that they are fully capable *alone* of radically curing many of the forms of syphilis. He notices with particular commendation the vapour and fumigating baths established by Mr. Green, of Great Marlborough street: they are said to be superior to any thing of the kind even in Paris.

In some cases, mercury fails in producing its proper effects upon the system. In such instances the hints of Dr. John Wilson, in our Journal for June last, upon the associated use of oil of turpentine and mercury, are worthy of attention.

Our opinion of this work may be very briefly stated. It is decidedly the most practically useful that has yet been published upon the subject of syphilis. We state this confidently; and we are quite sure that they who will peruse it with the attention which we have devoted to it will come to the same conclusion. Mr. Bacot has completely and satisfactorily effected the very laudable object he had in view, of saving the time of the surgical student by presenting him the result of the labours of previous writers, together with many interesting critical comments upon their opinions, which his practical experience and ability so well qualify him to offer. There is an attractive fluency and easiness in the style in which the work is written, which carry the reader along without the fatigue which arises from an application to most books that are worthy of deliberate study.

One omission Mr. Bacot should remedy in a future edition: there is neither an index nor a table of reference, and but few even of the chapters are headed with titles, to inform the reader of the subjects discussed in them.

